# Impact of Atrazine Exposure on the Microbial Community Structure in a Brazilian Tropical Latosol Soil

**DOI:** 10.1264/jsme2.ME19143

**Published:** 2020-04-09

**Authors:** Ana Flavia Tonelli Fernandes, Ping Wang, Christopher Staley, Jéssica Aparecida Silva Moretto, Lucas Miguel Altarugio, Sarah Chagas Campanharo, Eliana Guedes Stehling, Michael Jay Sadowsky

**Affiliations:** 1 Faculty of Pharmaceutical Sciences of Ribeirão Preto, University of São Paulo, Café Avenue s/n, Ribeirão Preto, SP 14040–903, Brazil; 2 Biotechnology Institute, University of Minnesota, 1479 Gortner Avenue, 55108 Saint Paul, MN 55108, USA; 3 Department of Soil Science ESALQ, University of São Paulo, 11 Pádua Dias Avenue, Piracicaba, SP 13418–260, Brazil; 4 Department of Soil, Water, & Climate, 1991 Upper Buford Circle and Department of Plant and Microbial Biology, 1479 Gortner Avenue—University of Minnesota, Saint Paul, MN 55108, USA

**Keywords:** 16S rRNA gene sequencing, qPCR, *atz*A, *atzD*, *trzN*

## Abstract

Atrazine is a triazine herbicide that is widely used to control broadleaf weeds. Its widespread use over the last 50 years has led to the potential contamination of soils, groundwater, rivers, and lakes. Its main route of complete degradation is via biological means, which is carried out by soil microbiota using a 6-step pathway. The aim of the present study was to investigate whether application of atrazine to soil changes the soil bacterial community. We used 16S rRNA gene sequencing and qPCR to elucidate the microbial community structure and assess the abundance of the atrazine degradation genes *atz*A, *atz*D, and *trz*N in a Brazilian soil. The results obtained showed that the relative abundance of *atz*A and *trz*N, encoding triazine-initiating metabolism in Gram-negative and -positive bacteria, respectively, increased in soil during the first weeks following the application of atrazine. In contrast, the abundance of *atz*D, encoding cyanuric acid amidohydrolase—the fourth step in the pathway—was not related to the atrazine treatment. Moreover, the overall soil bacterial community showed no significant changes after the application of atrazine. Despite this, we observed increases in the relative abundance of bacterial families in the 4^th^ and 8^th^ weeks following the atrazine treatment, which may have been related to higher copy numbers of *atz*A and *trz*N, in part due to the release of nitrogen from the herbicide. The present results revealed that while the application of atrazine may temporarily increase the quantities of the *atzA* and *trzN* genes in a Brazilian Red Latosol soil, it does not lead to significant and long-term changes in the bacterial community structure.

Pesticides have been a part of modern life since the 1940s (Gill and Garg, 2014). These compounds have positive effects on agricultural crops if used correctly, but failure to follow recommendations may cause environmental perturbation and have unintended consequences. Some chemical compounds, such as pesticides and herbicides, may undergo several transformation reactions, resulting in their transfer through environmental compartments, reaching ecosystems outside the application area, and sometimes resulting in effects on non-target organisms (Carvalho, 2017).

Brazil is one of the largest consumers of pesticides worldwide, in part due to its large agricultural production area and tropical climate (Luiz, 2015). The Brazilian agrochemical market grew by 190% between 2000 and 2010, while agrochemical sales increased by 13% in 2014 (IBGE, 2015; Luiz, 2015; IBAMA, 2016).

Atrazine, a herbicide in the s-triazine family, is an important component in maize production and is also widely used to control weeds in sorghum and sugarcane crops. Its chemical properties make the compound a possible contaminant of surface and groundwater due to its high susceptibility to leaching and above ground flow (Pratt *et al.*, 1998; Cavas, 2011; Fan and Song, 2014). Atrazine functions by inhibiting photosystem II (FSII), thereby operating as a suppressor of electron transport (Marin-Morales *et al.*, 2013; Luiz, 2015). Although atrazine is considered to only be moderately toxic, it has been reported to interfere with the ecosystem balance (Pratt *et al.*, 1998; Cavas, 2011). While the US Environmental Protection Agency (EPA) initially classified atrazine as a potentially carcinogenic class C chemical (United States Environmental Protection Agency, 2016), it was more recently shown to not cause cancer (Inoue-Choi *et al.*, 2016). While atrazine has also been reported to interfere with the homeostasis of estrogenic hormones (Ribaudo and Bouzaher, 1994; Moraes *et al.*, 2008; Carmo *et al.*, 2013), its role as a teratogen is contentious (Morgan *et al.*, 1996).

The persistence of herbicides in soil depends on dissipation processes including degradation, which may result in partial transformation or complete mineralization (Pelizzetti *et al.*, 1990; Balmer and Sulzberger, 1999; Carvalho, 2013). The main route of atrazine transformation in soil is via enzymatic, hydrolytic, displacement of substituents from the carbon atoms that form the s-triazine ring (Shapir *et al.*, 2007). The nitrogen released from atrazine catabolism is used by other soil microorganisms for growth and metabolism (Shapir *et al.*, 2007; Dutta and Singh, 2013). Atrazine mineralization involves six enzyme steps encoded by the *atz*ABC and *atzDEF* genes. The products of *atz*A, *atz*B, and *atz*C are responsible for the removal of chlorine and residues of isopropylamine and ethylamine in order to produce cyanuric acid (Sadowsky *et al.*, 1998; García-González *et al.*, 2003). In contrast, the *atz*DEF genes encode enzymes involved in the transformation of cyanuric acid to NH_3_ and CO_2_ (Shapir *et al.*, 2005; Shapir *et al.*, 2007; Porrúa *et al.*, 2010). Similar to *atz*A, the *trz*N gene in many Gram-positive bacteria encodes an enzyme belonging to the amidohydrolase superfamily, which participates in the transformation of atrazine into hydroxyatrazine (Mulbry *et al.*, 2002), the rate-limiting step in the biodegradation pathway.

The aim of the present study was to investigate whether the bacterial community structure in a Brazilian soil is influenced by exposure to atrazine. We used 16S rRNA gene sequencing to identify potential changes in the soil microbial community structure, qPCR to measure changes in the copy numbers of degradation genes following the exposure of soils to atrazine, and LC/MS-MS to assess the degradation of atrazine over time.

## Materials and Methods

### Field experiment and DNA extraction

A field plot experiment was conducted in the city of Tambaú, São Paulo, Brazil (21°31′S, 47°16′W, 540 meters) between November 2016 and February 2017. The local climate is humid subtropical (Cwa), with dry winters and rainy summers and temperatures ranging between 18–28°C (Peel *et al.*, 2007). Six mesocosms, each 9 m^2^, were constructed in an agricultural area with no history of atrazine use. These mesocosms were treated with atrazine to a final concentration of 6.5 Lha^–1^ (450‍ ‍mg kg^–1^). Soil at the site is a Red Latosol, which is characterized as having a clayey texture (Embrapa, 2013), pH of 5.7, and organic matter content of 2.3%. The experiment was conducted in triplicate and one sample was collected before the atrazine treatment. After the application of atrazine, samples were collected on weeks 1, 2, 3, 4, 8, and 12. In order to analyze the cultivable layer of the soil, samples were collected at a depth of 5 to 10‍ ‍cm from each mesocosm. The top layer of soil containing non-decomposed organic material, which may induce variations in microbial activity, was removed.

Total genomic DNA was extracted from soil samples using the PowerSoil^®^ DNA Extraction Kit (MoBio Laboratories). The integrity of DNA samples was assessed by 1% agarose gel electrophoresis, viewed, and photo-documented under ultraviolet light. An aliquot of each DNA sample was quantified by a fluorometric method using Qubit^®^ (Thermo Fisher Scientific).

### Abundance of atrazine degradation genes

Quantitative real-time PCR (qPCR) was performed using Roche LightCycler 480 II (Roche) and SYBR Green as a fluorescent dye. The atrazine degradation genes *atz*A, *atz*D, and *trz*N were quantified using the following primers: atzAf (5′-ACGGGCGTCAAT TCT ATGAC-3′), atzAr (5′-CACCCACCTCACCATAGACC-3′), atzDf (5′-TCCCACCTG ACATCAC AAAC-3′), atzDr (5′-GGGTCTCGAGGTTTGATTG-3′), trzNf (5′-CACCAGCACC TGTACGAAGG-3′), and trzNr (5′-GATTCGAACCATTCCAAACG-3′) (Devers *et al.*, 2004; Mulbry *et al.*, 2002). A standard curve was generated for each gene using a 10-fold serial dilution (30 copies 5‍ ‍μL^–1^ to 3,000,000 copies 5‍ ‍μL^–1^) of gBlocks^®^ Gene Fragments containing the target gene (IDTdna). Each assay was conducted with three replications for each standard, negative control, and DNA template. Amplification was performed in 20-μL reactions containing 10‍ ‍μL of 2×iTaq^TM^ Universal SYBR^®^ Green Supermix (Bio-Rad), 5‍ ‍μL of the DNA template or standard DNA, and a suitable volume of the forward and reverse primers. MgCl_2_ and BSA were used for the optimization of some genes. Nuclease-free water was added to bring the volume of the reaction to 20‍ ‍μL. Thermal cycling conditions are listed in the Supplementary material. All reactions were finished with a melting curve at 90°C for 5‍ ‍s and 60°C for 3‍ ‍s, and the cooling temperature was 40°C for 30 s.

qPCR efficiency (E) was calculated according to the equation E=[10^(–1/slope)^]–1. PCR reaction runs had an average efficiency of 94, 96, and 85% for *atz*A, *atz*D, and *trz*N, respectively. The R^2^ of all standard curves was ≥0.99. Controls without templates resulted in undetectable values in all samples.

### Bacterial community 16S rRNA sequencing

The V4 hypervariable region of the 16S rRNA gene was amplified from triplicate DNA extracts per sample using the 515F (5′-GTGCCAGCMGCCGCGGTAA-3′) and 806R (5′-GACTACHVGGGTWTCTAAT-3′) (Caporaso *et al.*, 2011) primer set and sequenced with the dual index method by the University of Minnesota Genomics Center (UMGC). Samples were paired-end sequenced on the Illumina MiSeq platform (Illumina) at a read length of 300 nucleotides (nt). Sequence data were processed and analyzed using mothur version 1.35.1 (Schloss *et al.*, 2009). Samples were trimmed to 150 nt and paired-end joined using fastq-join software with an average join length of approximately 10 nt, and then trimmed for quality based on the quality score (>35 over a 50-nt window), ambiguous bases (0), homopolymer length (≤8), and primer mismatches (≤2). High-quality sequences were aligned against the SILVA database version 123, subjected to a 2% pre-cluster, and UCHIME was used to remove chimeric sequences. Following quality trimming, the average sequence length was approximately 174 nt. In statistical comparisons, all samples were rarefied by random subsampling to 48,000 sequence reads per sample to reduce statistical bias due to varying numbers of sequences reads (Tomasek *et al.*, 2017). Operational taxonomic units (OTUs) were assigned at 97% similarity using complete-linkage clustering and taxonomic classifications were assigned against the Ribosomal Database Project, version 14. Different databases were used for alignment and OTU classification due to considerations previously described (Schloss, 2009).

### Atrazine detection in soil samples

Atrazine was extracted from 3‍ ‍g of each soil sample using 10‍ ‍mL of acetonitrile. Samples were vortexed for 1‍ ‍min and homogenized for 15‍ ‍min. Samples were then centrifuged at 4,000‍ ‍rpm at 4°C for 15‍ ‍min and 500‍ ‍μL of the supernatant was transferred to a 1.0-mL volumetric tube. The final volume was reached using ultra-purified water and this solution was filtered through a 0.20-μm membrane into an appropriate vial and injected into the analytical system. Atrazine was detected using liquid chromatography-tandem mass spectrometry (LC/MS-MS) as previously described (Fernandes *et al.*, 2018). Analyses were performed by the selected reaction monitoring (SRM) of protonated molecules [M+H]^+^ and their respective fragments. The transitions monitored (m/z) for atrazine were 216>174 and 216>146 (confirmation).

### Statistical analysis

ANOVA analyses with Tukey’s post-hoc test, Spearman’s rank correlations, and a canonical correspondence analysis were performed using XLSTAT version 2015.6. Shannon indices, beta diversity calculations, and ordination plots were calculated by mothur. A beta diversity analysis and ordination were performed using Bray-Curtis dissimilarity matrices (Bray and Curtis, 1957). Differences in community composition were evaluated by an analysis of molecular variance (ANOSIM) and sample clustering was evaluated by an analysis of molecular variance (AMOVA). Ordination was performed by a principal coordinate analysis (PCoA) and Spearman’s correlations of family abundance associated with ordination were calculated using the corr.axes command in mothur. Variance partitioning was performed by a partial redundancy analysis using the vegan package in R (R Core Team, 2012). All statistics were calculated at α=0.05 with Bonferroni corrections for multiple comparisons (Tomasek *et al.*, 2017).

### Accession number

Raw data were deposited in the Sequence Read Archive of the National Center for Biotechnology Information (NCBI) under BioProject accession number SRP123515.

## Results

### Assessment of *atz* gene abundance in soil

**Fig. 1.**
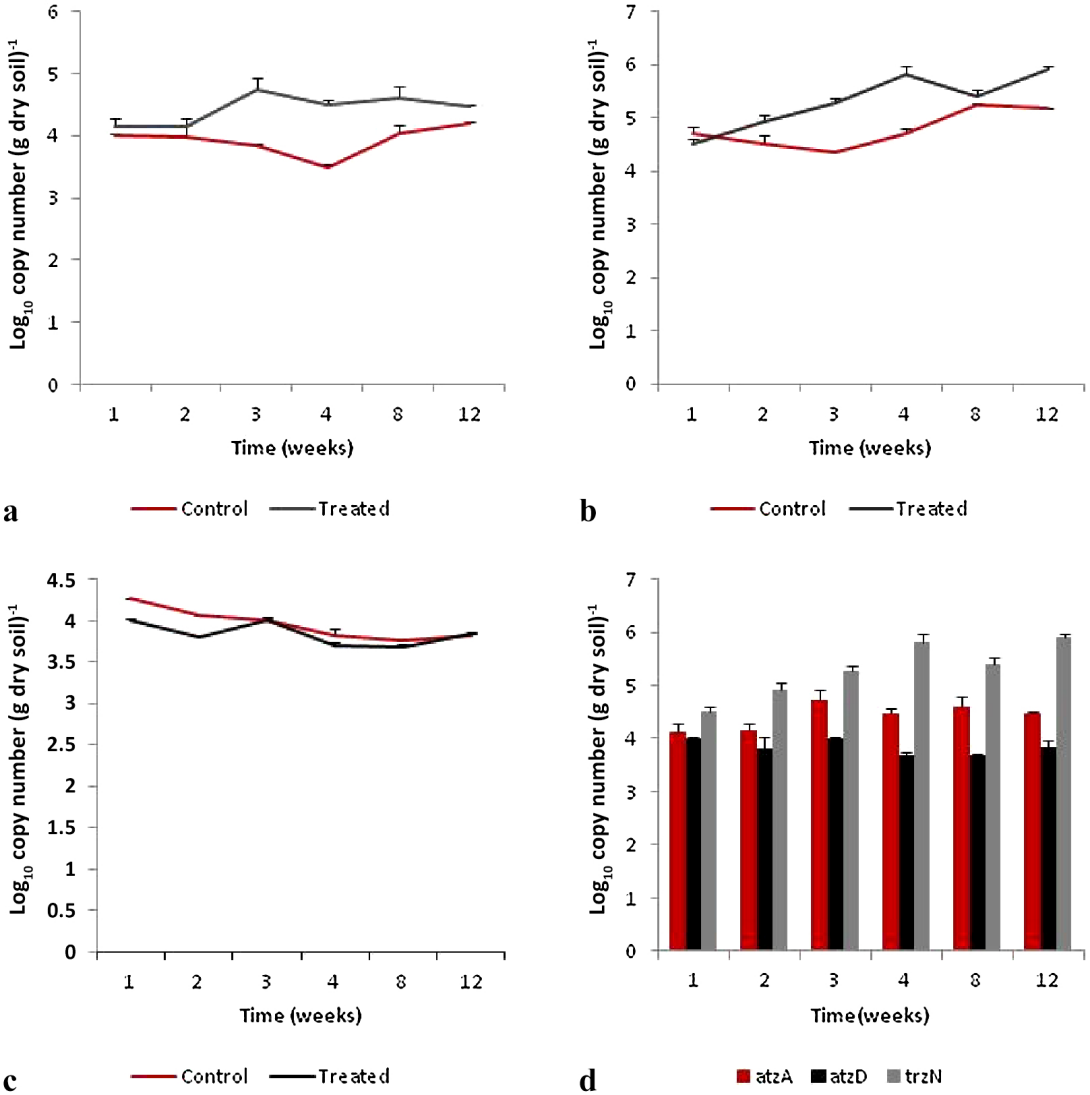
Abundance of *atz* genes after the application of atrazine to soil: **A**) *atz*A; **B**) *trz*N; **C**) *atz*D; and **D**) comparison between *atz*A, *trz*N, and *atz*D over time. Error bars reflect standard deviations (*n*=3).

Results from qPCR analyses revealed that the abundance of atrazine degradation genes responsible for initiating atrazine degradation (*atz*A and *trz*N) were not static and followed patterns of changes over time. The copy numbers of *atz*A and *trz*N increased in the first weeks after the application of atrazine. *atz*A gene abundance significantly increased by the third week. At the end of the experiment (by the 12^th^ week), *atz*A abundance values were close to those observed in the 1^st^ week (Fig. 1A). In contrast, the abundance of *trz*N changed from the 2^nd^ week, with greater values being observed in the 4^th^ week. At the end of the experiment, *trz*N in soil was still significantly higher than initial values (*P*<0.0001) (Fig. 1B).

Changes in the copy number of *atz*D in soil samples did not follow the same pattern observed for the *atz*A and *trz*N genes. The *atz*D copy number observed after the application of atrazine in soil was significantly lower than gene abundance in soil before its application (*P*<0.0001). Moreover, *atz*D gene abundance varied over the 12-week period (Fig. 1C). The present results also showed that *trz*N was significantly more abundant in soil after the application of atrazine than the *atz*A and *atz*D genes (Fig. 1D).

### Analysis of the soil bacterial community

The number of OTUs varied between 2,485 and 5,896 per sample, with a mean Good’s coverage of 96.4±1.2%, among all samples. Samples collected in the 2^nd^ and 8^th^ weeks after the application of atrazine to soil had significantly lower alpha-diversity (mean 6.82±0.14 and 6.88±0.12; *P*=0.0002), measured by the Shannon Index, than samples collected before its application (mean 7.21±0.06) (Table 1).

**Fig. 2.**
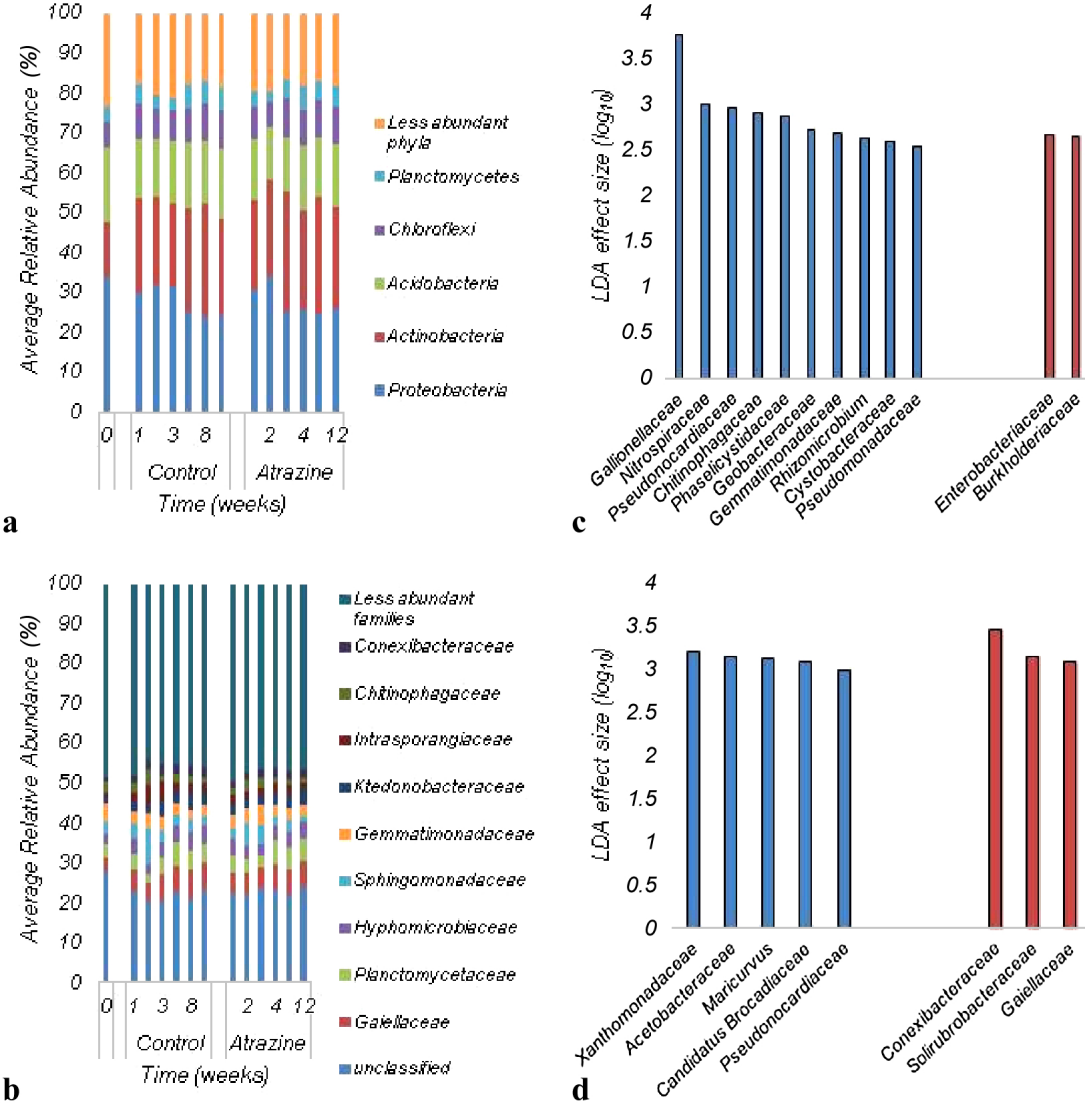
Distribution of microbial communities in soil samples collected before and after the application of atrazine: **A**) More abundant phyla; **B**) Classification at the family level. **C**) Linear discriminant analysis Effect Size of soil samples—4^th^ week; **D**) Linear discriminant analysis Effect Size—8^th^ week. Blue bars (**C** and **D**) represent samples collected before the application of atrazine and red bars (**C** and **D**) represent samples treated with atrazine.

The bacterial communities in soils during the 12-week sampling period after the application of atrazine were predominantly comprised of members of the phyla *Proteobacteria* and *Actinobacteria* (Fig. 2A). Fig. 2B shows the distribution of bacterial communities in soil samples at the family level. Soil microbial communities were predominantly comprised of less abundant families, and it was not possible to assign approximately 20–30% of sequence reads at this taxonomic level.

**Fig. 3.**
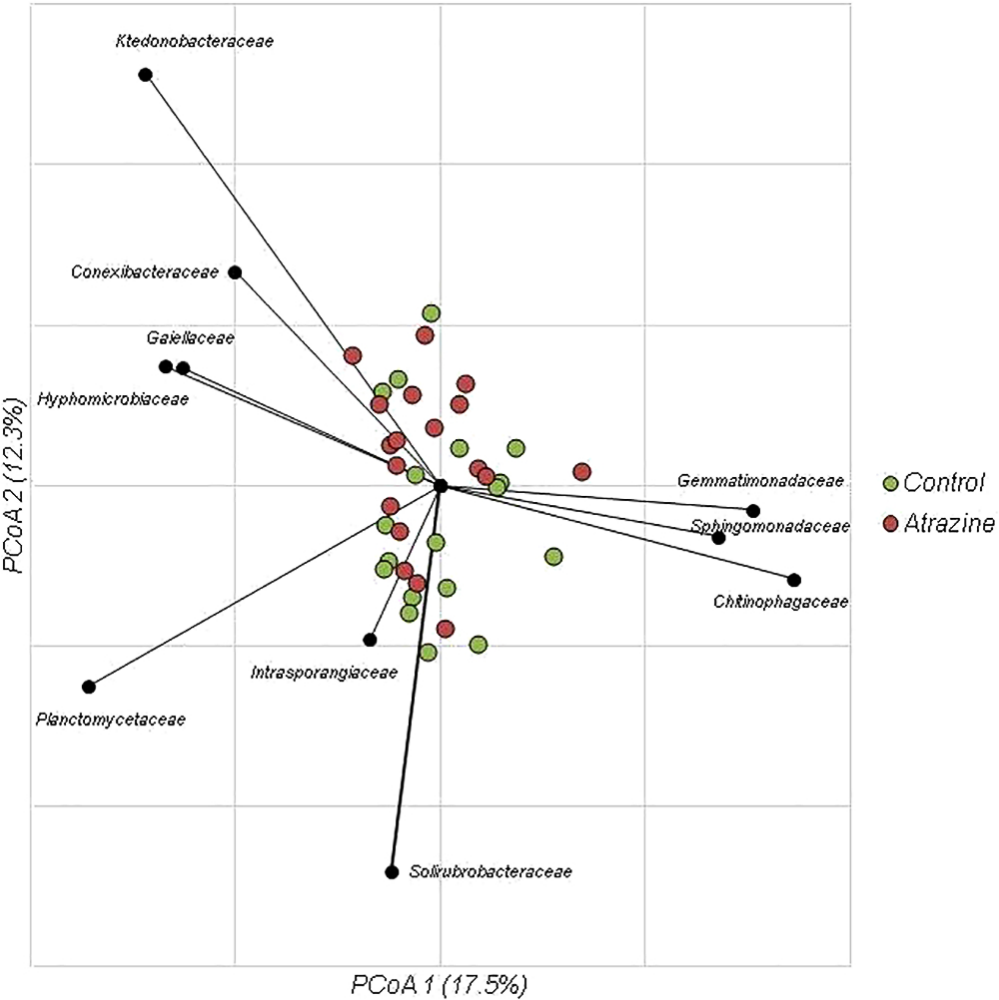
Principal coordinate analysis of Bray-Curtis distances (r^2^=0.72). The relative abundance of families shown correlated with axis positions via Spearman’s correlation (*P*<0.05).

While the bacterial community composition significantly differed among all soil samples tested (ANOSIM R=0.2362, *P*<0.0001), no significant difference was observed between soil samples collected before and after the application of atrazine. Moreover, the ordination of Bray-Curtis dissimilarity matrices by PCoA revealed that there was no significant difference between control and treated samples (Fig. 3), and sample clustering did not appear to be related to the atrazine treatment. Despite this, significant differences were observed in the relative abundance of families in the 4^th^ and 8^th^ weeks of incubation. Specifically, the abundance of *Enterobacteriaceae* and *Burkholderiaceae* increased in the 4^th^ week after the application of atrazine (Fig. 2C), while the abundance of *Conexibacteraceae*, *Solirubrobacteraceae*, and *Gaiellaceae* also increased by the 8^th^ week (Fig. 2D).

### Atrazine detection in soil

**Fig. 4.**
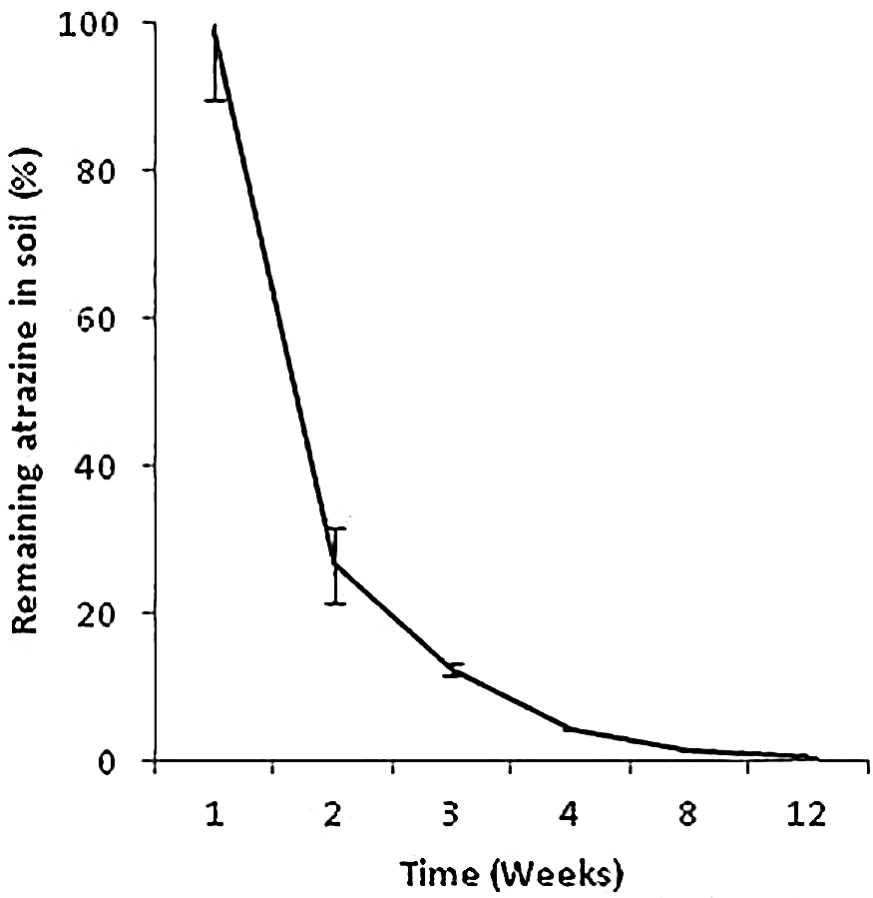
Monitoring atrazine degradation during 12‍ ‍weeks after herbicide application (% of initially applied atrazine) in soil with no history of atrazine use. Error bars reflect standard deviations (*n*=3).

LC/MS-MS analyses showed rapid atrazine degradation starting in the first weeks of treatment. A significant decrease in atrazine was observed between the 1^st^ and 2^nd^ weeks and also between the 2^nd^ and 8^th^ weeks (*P*<0.0001). After 2‍ ‍weeks, approximately 30% of atrazine remained in soil and less than 1% was detected in soil after 12‍ ‍weeks of application (Fig. 4). Atrazine was not detected in samples collected before the application of atrazine.

## Discussion

Quantification of the abundance of atrazine degradation genes, as a proxy for the atrazine-degrading soil microbiota, is important for assessing the genetic potential of a given bacterial community to degrade atrazine (Fajardo *et al.*, 2012; Martin-Laurent *et al.*, 2003). The quantification of soil microorganisms has several limitations, including the presence of viable, but non-culturable, bacteria and unknown species. Despite this, however, the qPCR technique is a reliable and sensitive method for detecting *atz* genes in soil environments (Thompson *et al.*, 2010) in the absence of requirements to culture microbes. Despite this, in the present study, the abundance of the *atz*A, *trz*N, and *atz*D genes was measured using qPCR and the results obtained showed that the *atz* gene, which encodes the enzyme responsible for initiating atrazine degradation, varied in response to the application of atrazine to soil. The copy numbers of *atz*A and *trz*N increased in the first week after the application of atrazine to soil, suggesting a rapid adaptation of the soil microbiota to atrazine even though the soil had no history of atrazine use. Furthermore, LC/MS-MS analyses showed that the most significant atrazine degradation occurred at the beginning of treatment, indicating that atrazine mineralization via atrazine chlorohydrolase was used and the genes detected in soil samples may be related to the degradation process. The natural attenuation of pollutants is sustained by the adaptation of the soil microbiota to pollutant biodegradation and is also essential for soil detoxification (Monard *et al.*, 2008). In this context, Monard *et al.* (2010) previously demonstrated that the *atz*A genes detected in soil samples were derived from indigenous soil bacteria rather than from inoculated strains, confirming the adaptation of the microbiota to atrazine in soil, and also that non-adapted soil may contain microorganisms capable of rapid atrazine degradation. Martin-Laurent *et al.* (2003) showed that the atrazine-degrading community rapidly increased in the presence of atrazine and may be regarded as an opportunistic microbiota. Furthermore, the atrazine degrader *Pseudomonas* was isolated from Amazon Forest soil with no history of pesticide use (Fernandes *et al.*, 2014). In contrast, Kersantè *et al.* (2006) suggested that a treatment with atrazine did not significantly affect *atz*A, *atz*B, or *atz*C gene abundance in soil samples with no bioaugmentation. However, these analyses were performed after 9‍ ‍d of the atrazine treatment, which may explain the divergence observed because the present results showed that *trz*N and *atz*A increased after 2 and 3‍ ‍weeks, respectively.

The *trz*N gene encodes an atrazine chlorohydrolase, analogous in function to AtzA. In the present study, qPCR analyses indicated that *trz*N was more abundant in soil samples treated with atrazine, than *atz*A and *atz*D. These results are consistent with the findings reported by Arbeli and Fuentes (2010) who identified *trz*N as the dominant gene in an atrazine-degrading community. Furthermore, Sagarkar *et al.* (2013) monitored *atz* genes during atrazine degradation in microcosm soil and showed that the *trz*N copy number increased in the second week in soil.

The AtzD gene is responsible for the degradation of cyanuric acid, an intermediate atrazine metabolite. According to qPCR results, its abundance in soil does not follow the same patterns of *atz*A and *trz*N, showing that *atz*D is not directly affected by the application of atrazine to soil. Cyanuric acid is a natural product that is widespread in nature and was detected in soil samples before the usage of atrazine in agricultural practice. This compound is not only the result of atrazine degradation (Fruchey *et al.*, 2003), it may be degraded by several enzymes.

The sequencing of 16S rRNA genes via the Illumina platform revealed that the bacterial community structure did not significantly change following the application of atrazine to soils. Treated soil samples showed high alpha diversity, even after a slight decrease in the 2^nd^ and 8^th^ weeks of incubation. Atrazine biodegradation in soil has been extensively studied and is linked to high bacterial diversity. Several bacterial strains have already been described as atrazine-degrading microorganisms (Martin-Laurent *et al.*, 2003). Herbicides may inhibit some microorganisms due to toxicity, but also stimulate specific groups because some herbicides, such as atrazine, are a nutrient source (Ribaudo and Bouzaher, 1994; Bonfleur *et al.*, 2015). Moreno *et al.* (2007) reported an increase in the microbial biomass after a treatment with atrazine and suggested that variations in the soil community reflect the ability of microorganisms to respond to atrazine and adapt. Voets *et al.* (1974), using culture-based techniques, showed that long-term atrazine treatments induced significant changes in the soil microbial population. However, while the total number of bacteria and fungi did not decrease, specific groups of microbes were reduced. Moreover, Godoi *et al.* (2014) used fluorescence *in situ* hybridization (FISH) to examine Brazilian soil samples and found that samples treated with atrazine showed less diversity. These findings indicate that it is difficult to compare pesticide effects on the soil microbiota due to the various experimental designs and techniques used. Nevertheless, sequence-based approaches, such as 16S amplicon analyses and metagenomics, are powerful tools for investigating microbial ecology on a greater scale than previously considered possible (Riesenfeld *et al.*, 2004) and are the best tools for examining soil microbial community responses to the addition of herbicides.

The bacterial community analysis performed herein revealed that the most abundant phyla in soil samples were *Actinobacteria* and *Proteobacteria*. These phyla were already shown to positively correlate with atrazine concentrations in soil and have been identified as the most abundant phyla in soils contaminated with pesticides (Liu *et al.*, 2016). Moreover, several atrazine degraders belong to these phyla (Smith *et al.*, 2005; Udiković-Kolic *et al.*, 2010). Relative abundance analyses also indicated that members of the families *Enterobacteriaceae* and *Burkholderiaceae* significantly increased in samples collected after four weeks of atrazine application. These families include Gram-negative microbes that may possess the *atz*A gene, which increased from the 3^rd^ week. Similarly, members of the families *Conexibacteraceae*, *Solirubrobacteraceae*, and *Gaiellaceae* increased in the 8^th^ week and may contain the *trz*N gene, which is mostly found in Gram-positive bacteria and indicates the presence of *Actinobacteria* in soil (Sagarkar *et al.*, 2013). These families have not yet been associated with atrazine degradation in soil; however, limited information is currently available on these microorganisms and their role in the environment. While the family *Conexibacteraceae* only contains one species and *Conexibacteraceae* and *Solirubrobacteraceae* only comprise a few culturable species, many 16S rRNA gene sequences of soils from different regions of the world have high similarities to culturable organisms from this group (Whitman *et al.*, 2012). The family *Gaiellaceae* is a novel and poorly studied family comprised of strict aerobes. Members of this family have been associated with plants and correlated to the ratio of soil carbon to nitrogen (Hermans *et al.*, 2016). Therefore, a more detailed understanding of soil bacteria, at various taxonomic levels, is needed in order to clarify the effects of contaminants on the structure of soil bacterial communities.

In conclusion, results from the present study revealed the capacity of the soil microbiota of a Brazilian Red Latosol to rapidly adapt after an initial exposure to a herbicide, and the application of atrazine may increase the abundance of the *atz*A and *trz*N genes. Furthermore, since the bacterial community structure does not significantly change over time after the application of atrazine, the increased relative abundance of some families may be related to the abundance of the *atz*A and *trz*N genes.

## Citation

Fernandes, A. F. T., Wang, P., Staley, C., Aparecida Silva Moretto, J., Miguel Altarugio, L., Chagas Campanharo, S., et al. (2020) Impact of Atrazine Exposure on the Microbial Community Structure in a Brazilian Tropical Latosol Soil. *Microbes Environ ***35**: ME19143.

https://doi.org/10.1264/jsme2.ME19143

## Supplementary Material

Supplementary Material

## Figures and Tables

**Table 1.** Alpha diversity indices (mean±standard deviation) for triplicate soil samples. Values sharing the same letter did not significantly differ by Tukey’s *post-hoc *test (*P*>0.05).

**Treatment**	**Time (weeks)**	**Shannon**	ACE*
	0	7.21±0.07 A	5649±923 AB
Control	1	7.28±0.06 A	7450±789 AB
	2	7.07±0.13 ABC	6040±1208 AB
	3	7.23±0.13 A	5925±1415 AB
	4	7.26±0.05 A	10159±501 A
	8	7.20±0.03 AB	9897±534 A
	12	7.23±0.05 A	8668±1895 AB
Atrazine	1	7.22±0.06 A	5686±354 AB
	2	6.82±0.14 BC	5031±1496 AB
	3	7.10±0.22 ABC	7837±1963 AB
	4	7.24±0.06 A	9670±1229 A
	8	6.89±0.12 BC	6108±3461 AB
	12	7.13±0.09 ABC	9111±1722 AB

* *ACE: abundance-based coverage estimate.*
