# Supplementary material for: Impact of Atrazine Exposure on the Microbial Community Structure in a Brazilian Tropical Latosol Soil
Source: Microbes Environ. 2020 Apr 9;35(2):ME19143. doi: 10.1264/jsme2.ME19143 (PMC7308567; doi:10.1264/jsme2.ME19143)
Supplement: Supplementary file 1 — Supplementary Material [file 35_19143_s1.pdf]

Supplementary Material

Table S1. Conditons for qPCR reactions for *atz*A and *atz*D genes.

Pre-incubation	Denaturation	Annealing	Extension	Signal aquisition
50˚C - 2 min	95°C	60˚	72°C	81˚C
95˚C - 5 min	15 sec	30 sec	30 sec	15 sec
	40 cycles			

Table S2. Conditions for qPCR reactions for *trz*N.

Pre-incubation	Denaturation	Annealing	Extension
50˚C - 2 min	95°C	55˚	72°C
95˚C - 5 min	45 sec	45 sec	45 sec
	45 cycles		
